# Prior exposure of *Arabidopsis* seedlings to mechanical stress heightens jasmonic acid-mediated defense against necrotrophic pathogens

**DOI:** 10.1186/s12870-020-02759-9

**Published:** 2020-12-07

**Authors:** Eric Brenya, Zhong-Hua Chen, David Tissue, Alexie Papanicolaou, Christopher Ian Cazzonelli

**Affiliations:** 1 Hawkesbury Institute for the Environment, Western Sydney University, Locked Bag 1797, Penrith, NSW 2751 Australia; 2 Present address: Department of Biochemistry, Cellular and Molecular Biology, University of Tennessee, Hesler Biology Building. 1441 Circle Drive, Knoxville, TN 37996 USA; 3 School of Science, Western Sydney University, Penrith, NSW 2751 Australia

**Keywords:** Mechanical stress, Necrotrophic pathogen, Jasmonic acid, Stress priming, *Alternaria brassicicola*, *Botrytis cinerea*

## Abstract

**Background:**

Prolonged mechanical stress (MS) causes thigmomorphogenesis, a stress acclimation response associated with increased disease resistance. What remains unclear is if; 1) plants pre-exposed to a short period of repetitive MS can prime defence responses upon subsequent challenge with necrotrophic pathogens, 2) MS mediates plant immunity via jasmonic acid (JA) signalling, and 3) a short period of repetitive MS can cause long-term changes in gene expression resembling a stress-induced memory. To address these points, 10-days old juvenile *Arabidopsis* seedlings were mechanically stressed for 7-days using a soft brush and subsequently challenged with the necrotrophic pathogens, *Alternaria brassicicola,* and *Botrytis cinerea*. Here we assessed how MS impacted structural cell wall appositions, disease symptoms and altered gene expression in response to infection.

**Results:**

The MS-treated plants exhibited enhanced cell wall appositions and jasmonic acid (JA) accumulation that correlated with a reduction in disease progression compared to unstressed plants. The expression of genes involved in JA signalling, callose deposition, peroxidase and phytoalexin biosynthesis and reactive oxygen species detoxification were hyper-induced 4-days post-infection in MS-treated plants. The loss-of-function in JA signalling mediated by the JA-insensitive *coronatine-insensitive 1* (*coi1*) mutant impaired the hyper-induction of defense gene expression and promoted pathogen proliferation in MS-treated plants subject to infection. The basal expression level of *PATHOGENESIS-RELATED GENE 1* and *PLANT DEFENSIN 1.2* defense marker genes were constitutively upregulated in rosette leaves for 5-days post-MS, as well as in naïve cauline leaves that differentiated from the inflorescence meristem well after ceasing MS.

**Conclusion:**

This study reveals that exposure of juvenile *Arabidopsis* plants to a short repetitive period of MS can alter gene expression and prime plant resistance upon subsequent challenge with necrotrophic pathogens via the JA-mediated *COI1* signalling pathway. MS may facilitate a stress-induced memory to modulate the plant’s response to future stress encounters. These data advance our understanding of how MS primes plant immunity against necrotrophic pathogens and how that could be utilised in sustainable agricultural practices.

## Background

Mechanical stress induced by stamping on juvenile crops has been utilized for centuries by farmers in China and Japan in a practice called *mugifumi* to harden crops against biotic and abiotic stresses [1]. Prolonged MS induced by wind or touch can alter the plant cell wall by enhancing elastic resilience and flexural stiffness, culminating in a plant with shorter stature, reduced leaf size, and increased tensile strength; this acclimation response is known as thigmomorphogenesis [2, 3]. Prolonged MS-induced lignin, cellulose, phenylalanine ammonia-lyase, peroxidases, and reduced intracellular spaces to make it harder for pathogens to invade the plant cell [4–8]. A single event of MS from exogenous water spray to *Arabidopsis* seedlings growing in artificial media or the bending of leaves from soil grown plants, has been reported to cause large-scale differential gene expression of 10 and 2.5% respectively, in naïve *Arabidopsis* tissues [9, 10]. The majority of MS-responsive genes were implicated in cell wall modifications, hormone regulation, organelle, and calcium signalling, that collectively mediate stress acclimation responses in plants [9–12]. In the absence of MS, the expressions of most MS-responsive transcripts returned to their basal level of expression. However, some genes such as *TOUCH* transcripts (*TCH3* and *TCH4*) involved in calcium signalling and cell-wall modification remained responsive to MS, while others became desensitized to subsequent stimulation [11, 12]. The rapid change in gene expression in response to a single stimulus of MS can provide a decreased sensitivity to prolonged MS and protect the plant from unnecessarily responding to repeated MS induced by wind or touch. The desensitisation of plants to repetitive MS could represent ‘a memory’ that enables them to cope with continued events of MS [13–15].

Plant immunity involves phytohormone biosynthesis and signalling, in particular JA has been extensively linked with plant defenses against necrotrophic infection. Naïve *Arabidopsis* plants subject to a transient 40 s of gentle leaf rubbing without overt tissue damage showed enhanced resistance to *Botrytis cinereal*, although this was independent of JA biosynthesis or signalling [8]. In contrast prolonged MS (14-days of touch) can enhance JA accumulation to induce thigmomorphogenesis, and also promoted resistance against *B. cinerea* in *Arabidopsis* [16]. Exogenous water spray will also transiently elicit a JA response that can last a few hours [9] and *Arabidopsis* mutants impaired in JA biosynthesis and signalling (*allene oxide synthase*, and *myc2 myc3 myc4* triple mutant) were shown to prevent thigmomorphogenesis and/or the expression of some MS-inducible genes*,* thus implicating JA in MS-induced responses [9, 16]. JA signalling is restrained by JASMONATE-ZIM-DOMAIN (JAZ) repressors that interact with the F-box protein COI1 (CORONATINE INSENSITIVE1), which is part of the SCF (Skp-Cullin-F-box) E3 ubiquitin ligase complex involved in the direct interaction with Jasmonyl-L-Isoleucine (JA-Ile)/Coronatine (COR) [17]. In response to stress, the COI1-JAZ co-receptor complex leads to the proteasome-dependent degradation of JAZ repressors and the release of MYC transcription factors that affect JA dependent processes. The loss-of-function mutation in *COI1* abolishes the formation of the protein complex and JA responses required for wound- and jasmonate-induced transcriptional regulation of plant defense [18]. Additional signalling pathways involving other hormones such as gibberellins also facilitate MS-induced thigmomorphogenesis and stress acclimation in plants [19, 20]. What remains unclear is if a short repetitive period of MS culminating in JA responses [9] can elicit defence against necrotrophic pathogen infection via a JA-dependent or JA-independent process.

Prolonged stress to plants, including salt stress, extreme temperature and pathogen infection can induce a ‘memory’ mediated by epigenetic processes to enhance the plants acclimation response to future stress encounters [21]. Reports indicate that MS-induced responses in *Arabidopsis* involve epigenetic modification such as histone methylation [22, 23]. However, whether MS application representing the first stress encounter by plant can induce stress memory or prime the plants’ response to subsequent stress (pathogens or abiotic stress) is unknown. Priming is defined as the enhanced sensitivity and responsiveness of plants to stress as a result of prior experience that leads to increased resistance or tolerance to biotic and/or abiotic stress [24]. Priming provides plants with a beneficial advantage to respond faster and mitigate a subsequent stress. The priming phase occurs at the physiological, transcriptional, proteomic, metabolic and epigenetic levels as a warning signal to mount a stronger response that can persist through the plants’ life cycle and in some cases can be inherited to the subsequent generation [24–26].

The effect of MS on plant defense has mostly been assessed utilising *B. cinerea* as the model necrotrophic pathogen via histochemical or phenotypic studies. However, the molecular mechanisms of MS-induced plant immunity, and if MS can prime defense responses, remains unclear. Here, we investigated if 10-days old juvenile *Arabidopsis* seedlings subject to 7-days of MS could prime defense against *A. brassicicola* infection. The genus *Alternaria* are important fungal pathogens that cause black spot disease in *Brassica* species, leading to a yield loss of more than 15% worldwide [27]. Infection by *Alternaria brassicicola* is characterized by dark brown lesions and a yellowish chlorotic halo surrounding a necrotic lesion on leaves [28]. *A. brassicicola* is a necrotrophic pathogen that requires mostly JA mediated defense and feeds on dead plant tissue in order to proliferate [29, 30].

We hypothesised that a short period of repetitive MS would enhance the plants’ tolerance or resistance for several days post-stimulation by altering phytohormone accumulation, enhancing defense gene expression and cell wall secondary metabolites that promote plant defense. Here we  show that plants subjected to a short period of repetitive MS primed a defense response against *A. brassicicola* in the WT, but not in the JA-insensitive mutant *coi1–16*, implicating JA in MS-induced immunity. Our study revealed that a non-chemical strategy such as MS can heighten plant defenses in seedlings and promote resistance against necrotrophic pathogens.

## Results

### A short period of MS induces defense-related metabolites

**Fig. 1:**
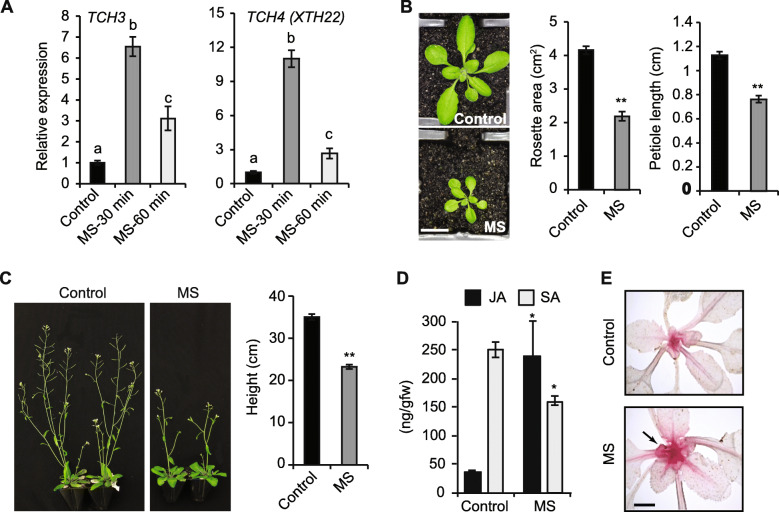
Effect of a short period of MS to juvenile *Arabidopsis* seedlings*.* **A** *TCH*-inducible gene expression within 30 and 60 min after 10 s of MS. Relative mRNA expression levels were normalized using *β-ACTIN* as the housekeeping gene (*n* = 3). **B** Petiole length and rosette leaf area in a 10-day old plant subject to 7-days of repetitive MS (touched twice daily for 10 s). A representative image of a 17-day old plant has been displayed and data was averaged from scoring individual plants (*n* = 15 plants). **C** Floral stem height and reproductive architecture in MS-treated and control plants. 10-day old plants were subjected to 7-days of MS without any further stimulation and allowed to flower. Phenotypes were scored in a 35-day old plant from the main floral bolt. A representative image has been displayed and the floral stem height defines the average of multiple independent plants (*n* = 15). **D** Jasmonic acid (JA) and salicylic acid (SA) levels were quantified in a whole rosette from juvenile plants subject to 7-days of MS. Leaves were harvested 30 min post-MS (*n* = 10 plants). **E** Leaves from controls and 7-days MS plants stained with phloroglucinol to highlight lignin accumulation as red colouration (indicated by arrow). Error bars show standard error of biological variation. Statistical significance denoted by letters (**A**) was determined using ANOVA with the Bonferroni test, and asterisks **(B**, **C**, **D)** determined with student’s t-test (*p* < 0.05). Scale bars = B, 1 cm; C, 5 cm; E, 1 cm

Here, we investigated if a 7-day period of repetitive MS to wild type (WT) *Arabidopsis* seedlings can alter defense metabolites and induce thigmomorphogenesis. A single event of MS using a soft brush (10 s twice daily, 8 h intervals) to 10-days old juvenile *Arabidopsis* plants elicited high expression of *TCH3* and *TCH4* (*XTH22*) genes, which are involved in calcium signalling and cell wall modifications, respectively [31, 32]. The gene expression lasted up to 60 min (3-fold higher) or perhaps longer reaching a maximum peak at 30 min following MS (6- to 10-fold) (Fig. 1A). The 7-days of MS caused a significant reduction in the overall plant growth, evident by a 32 and 47% decrease in petiole length and rosette area respectively, compared to the unstressed (control) plants (Fig. 1B). Interestingly, MS caused a 35% reduction in plant height despite MS being ceased before the emergence of the inflorescence stem (Fig. 1C). Therefore, a 7-day period of repetitive MS (herein referred to as a short period of MS) to juvenile seedlings induced thigmomorphogenesis in the absence of continued stimulation.

We next assessed MS effect on the accumulation of jasmonic acid (JA) and salicylic acid (SA) in MS and control plants using ultra-high-performance liquid chromatography-tandem mass spectrometry (UHPLC-MS/MS). Samples were collected 30 min after 7-days of MS when thigmomorphogenesis was prominently evident as a phenotype. MS significantly upregulated JA levels (~ 5-fold) and reduced SA (40%) in MS-treated compared to control plants, revealing an antagonistic effect between JA and SA signalling (Fig. 1D). MS-treated and control plants were stained with phloroglucinol that revealed high lignin deposition (reddish precipitation) in the shoot meristem and midrib of MS-treated plants perhaps indicating a higher tensile strength induced by MS (Fig. 1E). These data show that a short period of MS can promote the accumulation of metabolites that mediate thigmomorphogenesis and pathogen defense.

### Mechanical stress enhances resistance against *Alternaria brassicicola*

**Fig. 2:**
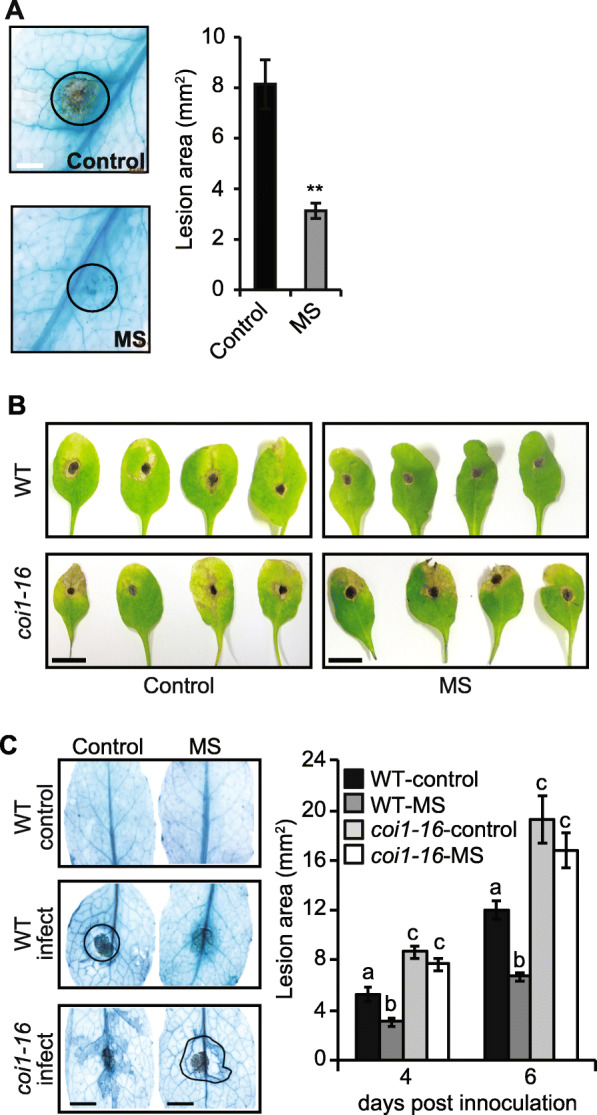
Defense response of MS plants to *B. cinerea* and *A. brassicicola* infection. The 5th to 7th true leaf from 17-day old plants were inoculated with 5 μL of 3.5 × 10^4^ spores/mL^− 1^ of pathogens (*n* = 15 plants)*.* **A** Lactophenol-trypan blue was used to stain fungal hyphae and to determine necrotic lesion denoted by circle in leaves from control and MS plants 48 hpi. A representative stained image and the average lesion area are displayed (*n* = 15 leaves). **B** Representative images of leaves from MS wild-type plant (WT) and the JA mutant *coi1* infected with *A. brassicicola* (5 dpi). **C** Necrotic lesions were stained with lactophenol trypan blue and the lesion area (mm^2^) denoted by circle with arrow was measured to provide a quantitative measurement of pathogen proliferation (*n* = 15). All data are representative of at least two independent experiments. Error bars show the standard error of biological variation. Statistical significance denoted by letters was determined using ANOVA with the Bonferroni test (*P* < 0.05). Scale bars = A, 0.2 cm B, 1 cm; C, 0.3 cm

We questioned if 7-days of MS could enhance plant defense upon subsequent challenge with *B. cinerea* or *Alternaria brassicicola*. Leaves from MS-treated plants (the 5th, 6th, and 7th true leaves) were inoculated with 5 μl of *B. cinerea* spores 30 min after the last stimulation. Leaves from MS-treated plants showed a reduced necrotic lesion area (3 mm^2^) confirmed by lactophenol-trypan blue (LPTB) stain compared to control plants (8 mm^2^) (Fig. 2A). Consistent with previous report [16], we show that a short 7-day period of MS can induce resistance (62.5% less in lesion area; 48 h post-inoculation) in *Arabidopsis* against *B. cinerea* infection.

Five days post-inoculation with *Alternaria brassicicola*, the control plants showed enhanced leaf chlorosis (yellowing of cells due to chloroplast degradation) and a halo of necrotic lesion surrounding the initial inoculation site, revealing an incompatible interaction [33]. The rosette leaves from MS-treated plants did not display obvious signs of chlorosis or the halo of necrotic lesion (Fig. 2B). Staining of leaves with lactophenol trypan blue revealed that the pathogen was restricted to the initial spore inoculation site in MS-treated leaves (Fig. 2C). In control plants, the pathogen appeared to degrade tissues surrounding the inoculation site (Fig. 2B). Four- and six-days post-inoculation, the lesion area was significantly larger in control plants (5.2 mm^2^ and 11.9 mm^2^) compared to MS-treated plants (3.0 mm^2^ and 6.7 mm^2^) (Fig. 2C). Therefore, a short period of MS can enhance defense against *Alternaria brassicicola*.

### Mechanical stress enhances cell wall compounds and primes plant defense

**Fig. 3:**
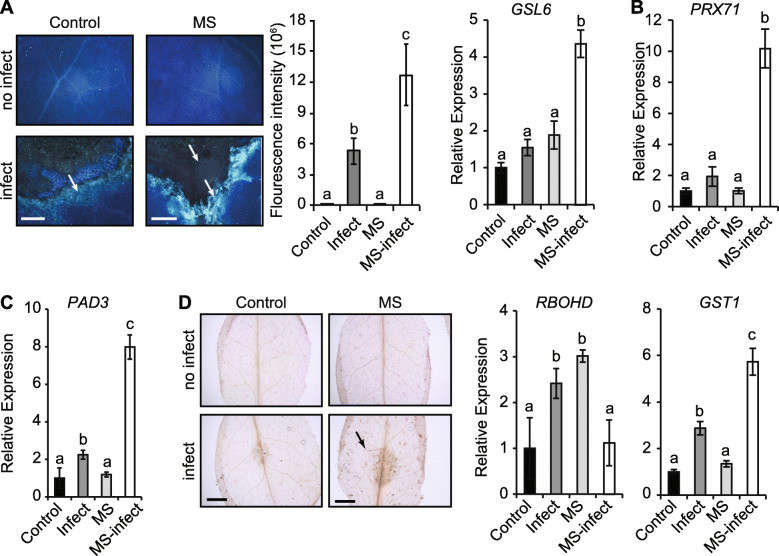
Changes in cell wall appositions and biosynthetic gene expression in response to *A. brassicicola* infection. **A** Leaves from control, infected, MS-treated and MS-infected plants were stained with aniline blue (3-dpi). A higher intensity of callose deposition fluoresces bright blue (indicated by arrow). The mean callose fluorescence intensity quantitatively determined in inoculated leaves. The image is a representation of several leaves (*n* = 15). The relative gene expression of *GSL6* was quantified 5 dpi. **B-C** The relative expression of *PRX71* and *PAD3* involved in the synthesis of lignin and the phytoalexin camalexin, respectively 5 dpi. **D** Leaves from control and MS -infected plants stained with 3,3′-Diaminobenzidine stain (36 hpi) that forms brown precipitation to indicate reactive oxygen species (ROS) accumulation. The relative expression of ROS marker genes (*GST1* and *RBOHD*), was determined 5 dpi. The expression levels of all genes were normalized to the *β-ACTIN* housekeeping gene (*n* = 4 biological reps) and are representative of at least two independent experiments. Error bars show standard error. Letters are statistical differences using ANOVA with the Bonferroni test (*p* < 0.05). Scale bars = A, 50 μm; C, 1 cm

Next, we probed whether MS enhances cell wall defenses (callose, lignin, camalexin, and ROS) to limit *A. brassicicola* proliferation. Analysis of callose which is a (1, 3)-β-glucan polymer that forms cell wall thickenings called papillae was enhanced in MS-infected leaves (> 2-fold deposition) compared to control-infected leaves. Callose deposition was confined to edges of the inoculation site in MS-infected leaves, whereas in control-infected leaves callose spread outside the inoculation site in areas of pathogen migration (Fig. 3A). Five days after inoculation, transcript levels of callose-associated marker gene *GLUCAN SYNTHASE–LIKE 6* (*GSL6*), was significantly upregulated (3.5-fold) in MS-infected compared to control-infected leaves (Fig. 3A). The *PEROXIDASE 71* gene (*PRX71*) which encodes cell wall-bound peroxidase that promotes lignification was significantly upregulated in MS-infected leaves compared to control-infected leaves (Fig. 3B). This correlated with the enhanced lignin in MS plants compared to control plants (Fig. 1E). In addition, the transcript level of *PHYTOALEXIN DEFICIENT 3* (*PAD3*) involved in phytoalexin (camalexin) biosynthesis, and defense against *A. brassicicola* [34] was significantly higher in MS-infected (8-fold) compared to control-infected (2.2-fold) plants (Fig. 3C). The increased lignin and callose deposition, as well as the hyper-induction of transcripts associated with camalexin, lignin and callose biosynthesis reveal that MS can enhance cell wall defenses to promote resistance against *A. brassicicola*.

MS increased ROS production as an early stress response to *A. brassicicola* infection in leaves- (36 h post-inoculation; hpi) (Fig. 3D). Five days post-inoculation, the expression of the ROS marker gene *RESPIRATORY BURST OXIDASE HOMOLOGUE D* (*RBOHD*; fine tunes the spatial control of reactive oxygen intermediates and hypersensitive response to the cell) [35] was significantly upregulated in control-infected plants (2.5-fold). However, *RBOHD* expression was not altered in the MS-infected leaves resembling a control level response (1-fold), despite the significant increase in the basal expression level of *RBOHD* in MS-plants (3.0-fold) (Fig. 3D). The transcript levels of *GLUTATHIONE S-TRANSFERASE 1* (*GST1*; scavenges and detoxifies ROS) were significantly hyper-induced in MS-infected leaves (6-fold) compared to control-infected (3-fold) plants, but unaffected in control or MS plants (Fig. 3D). Therefore, prior exposure of plants to repetitive MS and subsequent infection with pathogens may enhance the scavenging and detoxification of ROS, which might otherwise facilitate the virulence of necrotrophic pathogens [36, 37].

### JA mediates MS-induced resistance against *A. brassicicola*

MS enhances JA accumulation (Fig. 1D), which can mediate resistance against insect attack and necrotrophic pathogen infection [16, 34, 38]. Therefore, we examined whether MS-induced resistance against *A. brassicicola* required JA signalling. The JA-insensitive signalling mutant, *coi1–16*, has been demonstrated to be insensitive to exogenous application of MeJA and impairs jasmonate-induced signalling and transcriptional regulation of plant defences [18, 39, 40]. Therefore we investigated if the insensitivity of *coi1–16* to JA accumulation can likely impair the MS-induced resistance to *A. brassicicola* infection juxtaposed to the WT. Indeed, analysis of disease progression 5-days post-inoculation with *A. brassicicola* revealed that the *coi1–16* mutant was highly susceptible to infection with or without MS compared to the WT (Fig. 2B). The *coi1–16* plants showed enhanced leaf chlorosis and a larger necrotic lesion area (confirmed by staining with lactophenol trypan blue) surrounding the initial spore inoculation site when compared to WT (Fig. 2C). Therefore, a short period of MS to *Arabidopsis* seedlings can enhance defense against *A. brassicicola* infection via a JA-mediated signalling pathway.

### MS primes defense gene expression against *A. brassicicola* infection

**Fig. 4:**
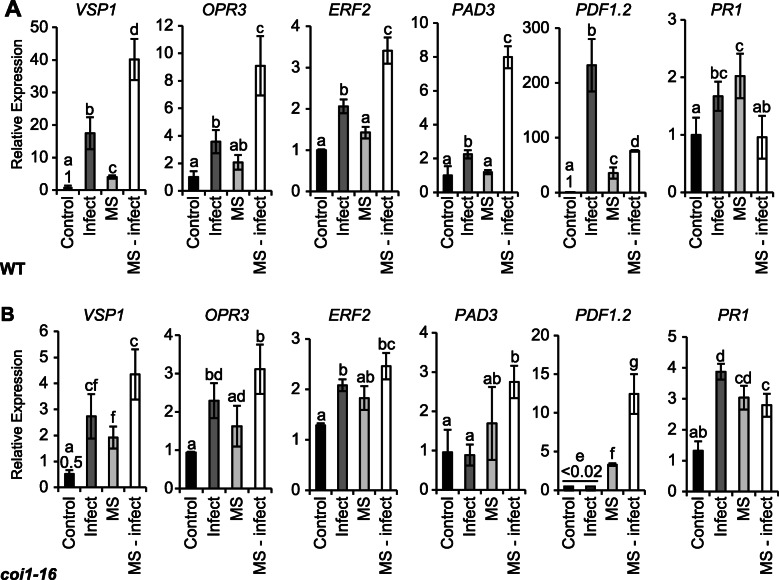
Regulation of defense gene expression in rosette leaves subject to MS and/or *A. brassicicola* infection. The 5th through 7th true leaves from control, infected, MS-treated and MS-infected 17-day old plants were inoculated with 5 μL of 3.5 × 10^4^ spores/mL^− 1^ of *A. brassicicola*. Gene expression was quantified 5 dpi. **A** Gene expression of *VSP1, OPR3*, *ERF3*, *PAD3*, *PDF1.2,* and *PR1* in WT. **B** Relative expression of *VSP1, OPR3*, *ERF3*, *PAD3*, *PDF1.2,* and *PR1* in *coi1–16* mutant compared to the WT. Relative gene expression levels were normalized to the *β-ACTIN* housekeeping gene. Error bars show standard error of the mean (*n* = 4 biological replicate). Letters are the statistical difference between treatments with Bonferroni test using a 2-way ANOVA, (*P* < 0.05). Statistical analysis in the *coi1–16* mutant (**B**) is relative to the WT (**A**). The experiment was repeated twice, with similar expression patterns obtained

We tested whether MS plants subsequently challenged with *A. brassicicola* would show altered gene expression indicative of priming triggered by the short period of MS (Fig. 4). Genes analysed included; *VEGETATIVE STORAGE PROTEIN 1* (*VSP1*; expression is induced by wounding and JA), *OXOPHYTODIENOATE-REDUCTASE 3* (*OPR3*; encodes a protein required for jasmonate biosynthesis), *ETHYLENE RESPONSE FACTOR 2* (*ERF2*; induced by JA and ethylene), *PLANT DEFENSIN 1.2* (*PDF1.2*; encodes an ethylene- and jasmonate-responsive plant defensin protein), as well as *PHYTOALEXIN DEFICIENT 3* (*PAD3*) and *PATHOGENESIS-RELATED GENE 1* (*PR1*; a marker gene induced by SA and indicative of a systemic acquired response). Five days post-inoculation, leaves from the control-infected plants showed enhanced expression of *VSP1* (17.5-fold), *OPR3* (3.8-fold), *ERF2* (2.1-fold), *PAD3* (2.1-fold), *PDF1.2* (232.2-fold) and *PR1* (1.8-fold) relative to those from control-uninfected plants (Fig. 4A). In MS-treated plants, the basal expression level of *OPR3*, *ERF2* and *PAD3* remained unchanged. Interestingly, the basal level of expression of *VSP1*, *PDF1.2,* and *PR1* remained constitutively higher (4.1-, 35.6-, and 2.1-fold, respectively) up to 5-days post-MS. MS-infected plants showed a significant hyper-induction of *VSP1* (40-fold), *OPR3* (9.1-fold), *ERF2* (3.4-fold) and *PAD3* (8-fold) compared control-infected plants (Fig. 4A). In contrast, *PDF1.2* and *PR1* expression was negatively regulated in MS-infected relative to control-infected tissues. That is; *PDF1.2* expression was reduced from 232.2 (control-infected) to 76.1-fold (MS-infected), while *PR1* expression was not significantly different to that of control plants (Fig. 4A). The hyper-induction or negative regulation of gene expression in MS-infected WT plants evidences that MS can prime a differential gene expression in response to subsequent pathogen infection.

We reasoned that a JA-mediated signalling pathway might facilitate the MS-induced priming of gene expression. The transcript levels of *VSP1*, *OPR3*, *ERF2, PAD3, PDF1.2,* and *PR1* were quantified in leaves from *coi1–16* plants compared to WT after 5 dpi when we observed more significant differences in pathogen infection (Fig. 2B and C). The basal expression level of *VSP1* (0.5-fold) and *PDF1.2* (0.01-fold) were reduced in control *coi1–16* (Fig. 4B) compared to WT plants (Fig. 4A). The expression of *VSP1* (2.7-fold), *OPR3* (2.2-fold), *ERF2* (2.5-fold) and *PR1* (3.9-fold) were upregulated in *coi1–16* infected tissues. Contrasting gene expression patterns between WT and *coi1–16* infected tissues revealed that *PDF1.2* and *PAD3* expression were no longer induced in *coi1–16*-infected tissues. The induction of *VSP1* expression was reduced (17.5- to 2.7-fold), while the induction of *PR1* expression was slightly higher (1.8- to 3.9-fold) in *coi1–16* compared to the WT (compared Fig. 4A to B). Interestingly, 5-days after ceasing MS, *coi1–16* MS plants showed an increase in the basal level of expression of *VSP1* (2-fold) and *PDF1.2* (3.3-fold) although significantly less compared to the WT except *PR1* (3-fold) which showed similar expression to the MS-treated WT plants (compare Fig. 4B and A). The hyper-induction of some gene expressions (e.g. *VSP1*, *OPR3*, and *ERF2*) observed in MS-infected tissues of WT (Fig. 4A), was not evident in MS-infected tissues of *coi1–16* (Fig. 4B). The induction of *PAD3* (2.7-fold) and *PDF1.2* (13.5-fold) was apparent in *coi1–16* MS-infected plants albeit significantly lower (> 2.5-fold less) compared to MS-infected WT plants (compare Fig. 4A and B). The MS actually caused the hyper-induction of *PDF1.2* expression in *coi1–16* infected plants (15-fold), a contra-regulation to reduction that was observed in MS-infected WT plants (compare Fig. 4A and B). Similarly, the induction of *PR1* expression in MS-infected *coi1–1*6 plants (2.8-fold) was reduced in MS-infected WT plants (0.9-fold) (compare Fig. 4A and B). Therefore, JA signalling mediated by *COI1* appears to prime the hyper-induction and/or contra-regulation of gene expression in MS-treated plants upon challenge with *A. brassicicola*.

### MS altered defense gene expression in naïve cauline leaves

**Fig. 5:**
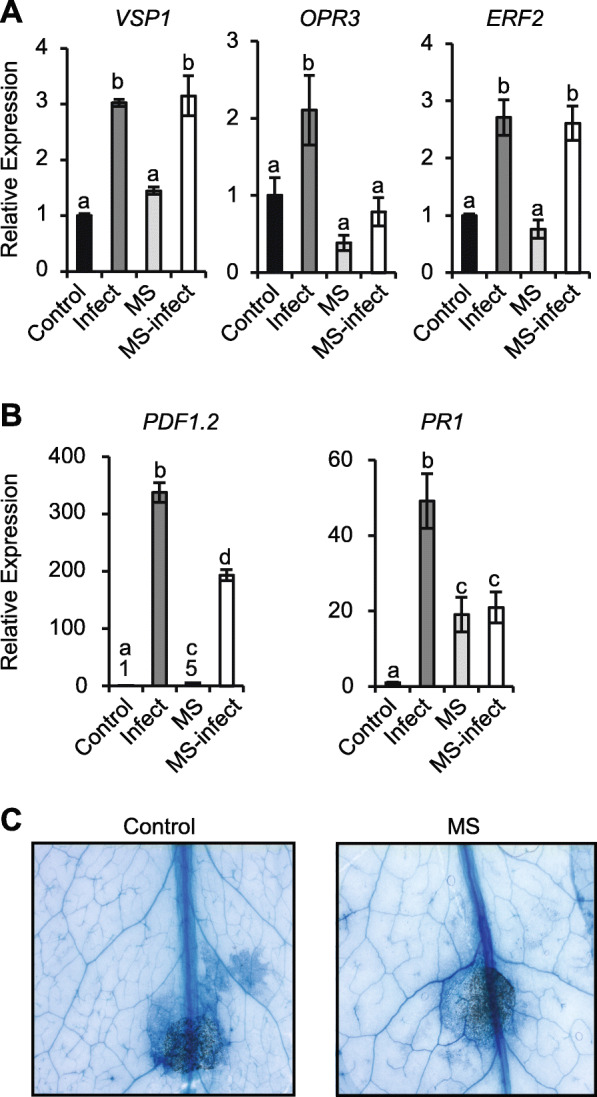
Analysis of defense gene expression and disease symptoms in naïve cauline leaves challenged with *A. brassicicola*. Ten days old WT plants MS for 7-days was allowed to grow without further stimulation until 2–3 cauline leaves emerged from the primary floral stem. The 1st and/or 2nd cauline leaves from MS-treated and control plants were inoculated with 5 μL of 3.5 × 10^4^ spores/mL^− 1^ of *A. brassicicola*. **A**, **B** The relative expression of pathogen-related defense genes in cauline leaves from control, and MS plants, 2 dpi. **C** Three days post-inoculation, cauline leaves were stained with lactophenol trypan blue to reveal the necrotic lesion area. Representative cauline leaves are displayed from two experiments showing similar results. Gene expression levels were normalized to the *β-ACTIN* housekeeping gene. Error bars show the standard error of the mean (*n* = 4). Letters are statistical differences between treatments with Bonferroni test using ANOVA, *P* < 0.05. The experiment was performed twice with similar results

The reduction in floral stem height observed in MS-treated plants several days post-stimulation (Fig. 1B), prompted us to test if naive cauline leaves that differentiate from the inflorescence stem would also show an altered defense response to *A. brassicicola* infection. Gene expression analysis at 2 dpi showed significant upregulation of *VSP1* (3-fold), *OPR3* (2.3-fold), *ERF2* (2.7-fold), *PDF1.2* (337.1-fold) and *PR1* (49.2-fold) expression in control-infected cauline leaves (Fig. 5A-B), a trend similar to that observed in control-infected rosette leaves (Fig. 4A). The expressions of *VSP1*, *OPR3* and *ERF2* were similar in the cauline leaves from MS and control plants (Fig. 5A). Interestingly, the basal level of expression of *PDF1.2* (5.0-fold) and *PR1* (19.1-fold) was constitutively upregulated in cauline leaves from MS plants (Fig. 5A), a trend also observed in rosette leaves (Fig. 4A). The expression of *VSP1* and *ERF2* was similar in infected cauline leaves from control and MS plants (Fig. 5A), revealing that the hyper-induction observed in MS-infected rosette leaves (Fig. 4A) was not retained in naïve cauline leaves. In contrast, *PDF1.2* and *PR1* expressions were negatively regulated in infected cauline leaves from MS-treated versus control plants (Fig. 5B), a trend also observed in infected rosette leaves (Fig. 4A). Analysis of cell death by staining with lactophenol trypan blue did not reveal any significant difference in disease susceptibility in cauline leaves from MS and control plants (Fig. 5C). Although the pre-exposure of the juvenile seedlings to MS was not sufficient to prime resistance in cauline leaves against *A. brassicicola* infection, the short period of MS could alter defense gene expression in naive cauline tissues.

## Discussion

**Fig. 6:**
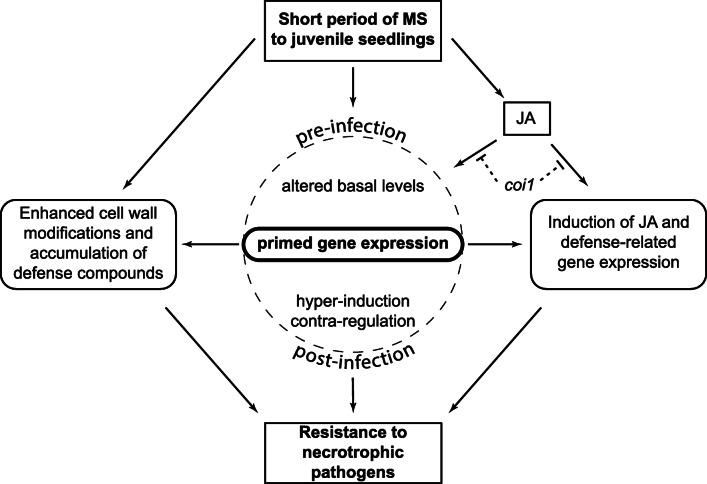
A model showing how prior exposure of juvenile seedlings to a short period of MS can prime gene expression and resistance to subsequent necrotrophic pathogen infection. The first stress encounter induced by repetitive MS (pre-infection) causes: 1) an increase in JA accumulation, 2) the accumulation of defense compounds (e.g. lignin and callose) that promote structural barriers, and 3) altered levels of basal gene expression. The secondary stress encountered by inoculating MS-treated plants with *A. brassicicola* causes the hyper-induction or contra-regulation of gene expression five-days post-infection that primes resistance to necrotrophic pathogen infection. A genetic perturbation in JA signalling mediated by the *coronatine-insensitive 1* (*coi1–16*) mutant affected the priming of gene expression and rendered MS-treated plants susceptible to infection

The application of MS twice daily to juvenile *Arabidopsis* plants for 7-days can enhance JA accumulation and facilitate thigmomorphogenesis. MS provided plants with an advantage to respond stronger or faster to subsequent challenge with *B. cinerea* and *A. brassicicola* infection by priming the hyper-induction and/or contra-regulation of gene expression associated with disease resistance. Here we discuss how MS induced defensive barriers and heightened JA signalling responses that trigger resistance against the infection by multiple necrotrophic pathogens and long lasting changes in gene expression indicative of the maintenance of epigenetic memory (Fig. 6).

### MS triggers defensive barriers to limit necrotrophic pathogen infection

MS-induced alterations in cell wall components can compensate for structural integrity challenges and contribute to plants first line of defense against pathogens [41–43]. For instance, MS enhanced the accumulation of callose, a (1,3)-*β*-glucan polymer that reinforces cell wall at *A. brassicicola* penetration site to limit infection (Fig. 3) [44]. This is consistent with a report showing callose accumulation in MS-strawberry leaf tissues challenged with *B. cinerea* [6]. Callose deposition correlated with the hyper-induction of *GSL6* gene expression in MS-infected leaves. Reports show that the overexpression of *Arabidopsis GSL5* promoted resistance during the early stage of powdery mildew infection in *Arabidopsis* [45, 46], and silencing of barley *HvGSL6* gene expression increased disease susceptibility to *Blumeria graminis* f.sp. *hordei* in barely [47]. Thus, the hyper-induction of *GSL6* expression and enhanced callose deposition in MS-infected tissues could limit pathogen penetration.

Lignin and camalexin are other cell wall structural compounds associated with promoting plant defence against pathogens. The *PRX71* gene encodes a cell wall-bound PEROXIDASE protein that functions to reduce cell expansion and cell wall damage, thereby limiting intracellular spaces for pathogen entry [48]. The overexpression of *PRX71* triggers lignification in *Arabidopsis* and increases resistance against *B. cinerea* [48–50]. Indeed, MS-treated leaves enhanced lignin deposition, and upon infection caused the hyper-induction of *PRX71* expression (Figs. 1 and 3). The *PAD3* gene encodes a cytochrome P450 enzyme that catalyses the conversion of dihydrocamalexic acid to camalexin, a compound that promotes resistance against pathogens [51, 52]. The loss-of-function mutation in *PAD3* enhances susceptibility to *A. brassicicola* in *Arabidopsis* [34], revealing that the hyper-induction of *PAD3* in MS plants could contribute to MS-induced resistance against *A. brassicicola* (Fig. 3). Therefore, the enhanced JA accumulation (Fig. 1D) and *PAD3* expression could synergistically contribute to disease resistance as previously reported [34, 53]. The oxidative stress-related gene *GLUTATHIONE S-TRANSFERASE 1* (*GST1*), which encodes for a protein that can catalyse the synthesis of glutathione-indole-3-acetonitrile, a precursor of camalexin biosynthesis [54] was also hyper-induced in MS-infected tissues (Fig. 3). Prior exposure of plants to MS and subsequent infection culminated in the hyper-induction of *PRX71*, *PAD3* and *GST1* expression, whose protein encoded products contribute to the biosynthesis of chemical compounds that promote resistance against *A. brassicicola* infection.

ROS accumulated in MS-treated leaf tissues subject to *A. brassicicola* infection (36 hpi of MS plants) (Fig. 3), consistent with previous reports in MS-treated strawberry and *Arabidopsis* leaves subjected to *B. cinerea* infection [6, 8]. ROS may facilitate the early signal transduction of MS responses, but may not be directly involved in MS-induced resistance. The loss-of-function in *RBOHD* and *RBOHF* genes were shown to impair ROS production [55], but were not involved in MS-induced defense against *B. cinerea* infection since MS-treated *rbohd* and *rbohf* mutants displayed a WT-like resistance to infection [8]. This reveals that the MS-induced resistance may be independent of ROS signalling. Exposure of plants to MS constitutively upregulated *RBOHD* expression up to 5-days post-stimulation. However, upon infection *RBOHD* expression in MS-treated plants was negatively regulated back to basal levels of expression, similar to the control (2.6-fold less) (Fig. 3). The reduced *RBOHD* expression in MS-infected plants could be due to the hyper-induction of *GST1* and *PRX71* expression, whose products are involved in ROS scavenging and hydrogen peroxide catabolic processes [56]. Thus, MS prior exposure of plants to repetitive MS may signal ROS limiting processes upon subsequent challenge with *A. brassicicola* to reduce cell death that could otherwise facilitate the virulence of necrotrophic pathogens [36, 37].

### JA signalling mediates MS-induced resistance against necrotrophic pathogen infection

A single event of soft MS by rubbing *Arabidopsis* leaves between the thumb and forefinger showed that JA may not be required for MS to mediate resistance against *B. cinerea* infection [8]. This is in contrast to a previous report that linked 4-weeks of MS-induced JA accumulation to *B. cinerea* resistance in *Arabidopsis* [16]. There could be differences in the acclimation response triggered by single versus prolonged MS. For example, a single event of MS [16] may transiently induce JA responses [9] that are not sufficient to induce thigmomorphogenesis [9] and elicit long-term JA-mediated responses. While, repetitive MS for days as shown here can enhance JA levels that culminate in growth changes in the plant and a long-lasting defense response [9, 16]. Here, we demonstrate that a short 7-day period of MS (twice daily during each photoperiod, for 10 s with an 8 h interval) with a soft brush can trigger thigmomorphogenesis, increase JA levels and promote resistance against *A. brassicicola* infection (Fig. 1).

JA is required to signal defense against necrotrophic pathogen infection [57]. For example, a prolonged period of MS shown to induce resistance in *Arabidopsis* to *B. cinerea* infection was impaired in the *opr3* mutant, and enhanced by the constitutive overexpression of *OPR3* [16]. Our result confirmed that the loss-of-function in COI1*,* an F-box protein essential for jasmonate responses and transcriptional regulation [18], compromised the MS-induced resistance to *A. brassicicola* infection (Fig. 2). The *coi1–16* mutant affected cell wall remodelling in *Arabidopsis* [58] and has a secondary mutation in *PENETRATION 2* (*PEN2*; implicated in resistance to pathogenic fungi) that can restrict pathogen growth in the cell periphery [59]. Thus, impairment in *coi1–16* may not only affect MS-induced JA resistance, but also the accumulation of cell wall components that impair the invagination of fungal hyphae within intracellular spaces [60]. We conclude that JA signalling via *COI1* mediates the MS-induced resistance against necrotrophic pathogen infection (Fig. 6).

*A. brassicicola* infection activated JA defense gene expression, keeping consistent with previous reports [29, 34]. An *Arabidopsis* genechip array showed that 12–36 h post-inoculation, before the onset of disease symptoms, many *A. brassicicola* induced genes were strongly increased in the WT plants compared to *coi1–16* plants. *VSP1* and *PDF1.2* were significantly reduced in *coi1* mutant, while *OPR3* involved in JA biosynthesis was found to be independent of *coi1* at the later stages of infection, keeping consistent with our data (compare Fig. 4) [34]. The regulation of gene expression in MS-treated plants at 5-dpi inoculation when there were visible differences in disease susceptibility was contrastingly different between WT and the *coil-16* mutant. The hyper-induction of *VSP1*, *OPR3, ERF2* and *PAD3* expression and/or contra-regulation of *PDF1.2* and *PR1* expression was either abolished or less evident in *coi1–16* compared to WT (Fig. 4). Consistent with a previous report [34], *PR1* was enhanced in *coi1* mutant indicating antagonistic effect of JA on *PR1* signalling via *COI1* pathway. The reduction of *PDF1.2* in the WT MS-infected rosette and cauline leaves compared to the WT control-infected plants revealed that MS negatively regulated *PDF1.2* expression upon pathogen infection via unknown mechanisms. A report indicates that *GLUTAREDOXIN 480* interaction with TGA factors can impair the expression of *PDF1.2* [61], but it is unknown if such mechanism occurs in response to MS. The basal expression level of *VSP1* and *PDF1.*2 in *coi1–16* mutant was constitutively reduced (> 50%) indicating that JA signalling via *COI1* is required to enable their expression and responses to MS. We conclude that JA signalling mediated through *COI1* facilitates the hyper-induction and/or altered regulation of gene expression in MS-infected plants (Fig. 6).

### MS primes defense responses upon subsequent pathogen infection

Previous exposure of juvenile *Arabidopsis* seedlings to a short period of MS enhanced the molecular response in adult plants to subsequent infection by *A. brassicicola* revealing that MS can prime defense against pathogens. MS-infected plants accumulated cell wall compounds that provided stronger defense barrier against pathogen invasion. Subsequent challenge of MS plants with *A. brassicicola* culminated in the hyper-induced expression of defense genes (*VSP1*, *OPR3*, *ERF2, PAD3*, *PRX71*, *GSL6* and *GST1*)  by approximately 2-fold, revealing that prior exposure of juvenile plants to MS can mount a stronger defense response and hence resistance to *A. brassicicola* infection. The response persisted in adult rosette leaves lasting for up to 4–5 days after MS had ceased, evident by the hyper-induction of defense gene expression. However, the primed defense response did not persist throughout the plants’ life cycle, since cauline leaves that emerged from MS-treated plants showed a WT-like susceptibility to infection (Fig. 5). The hyper-induction of *VSP1*, *OPR3* and *ERF2* gene expression was not observed in cauline leaves, yet the contra-regulation of *PDF1.2* and *PR1* gene expression apparent in rosette leaves was still evident in cauline leaves from MS plants, perhaps reflecting a stress-induced memory. Therefore, the short-term MS-induced resistance in rosette leaves was erased in naive cauline leaves in the absence of continued repetitive MS. The resetting of MS-induced memory would limit the energy cost associated in maintaining long-term immunity that might otherwise negatively affect growth and reproduction [24, 62].

Priming occurs at a physiological (hormonal changes), molecular (gene regulation), and/or epigenetic levels (DNA or histone modifications), essentially providing an acclimation strategy to heighten the plant’s defense capacity against future attack [24]. Prolonged drought, cold, salinity, light and pathogen infection have been reported to prime gene expression and strengthen the plant’s response to future stress encounters [21, 63–65]. We attribute the hyper-induction or contra-regulation of gene expression in MS plants to the higher endogenous JA levels, since the *coi1–16* JA signalling mutant impaired this heightened response (Fig. 6) [62].

### MS causes long-lasting changes in gene expression

MS is a ubiquitous stress stimulus perceivable by plants during development. Unstressed and naïve plants were proposed to maintain ‘memory’ of repetitive MS to prevent unwanted thigmomorphogenesis [14, 15, 66]. Our data demonstrates that pre-exposure of a plant to a short period of MS can constitutively upregulate the basal level of *RBOHD, VSP1*, *PDF1.2* and *PR1* expression for up to 5 days post-stimulation (Fig. 4). The upregulation of *PDF1.2* and *PR1* remained evident in naive cauline leaves that had not yet developed when MS was ceased > 10 days early before any sign of floral stem emergence from the adult rosette. Cells within the shoot apical meristem of juvenile seedlings remain in an undifferentiated state until the plant transitions from vegetative to reproductive stage, where the inflorescence meristem develops around 14 days after germination [67]. Thus, the altered level of gene expression in cauline leaves reveal that a short period of MS may have created a ‘stress memory’ in the shoot meristem of juvenile plants that was mitotically inherited through cell division into the inflorescence meristem [24–26]. The cauline tissues would have differentiated from specialised cell types within the inflorescence meristem during reproductive development and have retained some memory evident by the constitutively higher basal expression level of some genes. Future work on whether MS induced resistance in leaves can systemically be transferred to the root, a critical organ exposed to pathogens, symbionts and mechanical stress as it navigates through the soil will help us further understand the overall effect of MS on plant immunity.

Juvenile seedlings have the plasticity to respond to stress in their growing environmental; such as when a prolonged period of cold exposure (e.g. vernalization) triggers the early flowering of adult *Arabidopsis* plants mediated by histone modifications [68]. Stress-induced memory can persist in plants via epigenetic processes (histone modifications or DNA methylation) to alter a plants’ response to future encounters and in some cases can be inherited [21, 65]. For instance, the chromatin-modifying enzyme, SET DOMAIN GROUP 8 (SDG8), promotes permissive histone lysine methylation surrounding *TOUCH*, pathogen and flowering-time responsive genes and plays a key role in promoting vernalization as well as thigmomorphogenesis [22, 69, 70]. Further investigation is required to elucidate the mechanisms by which a short period of MS to 10 days old juvenile seedling can alter the basal levels and/or contra-regulate gene expression in rosette and naïve cauline leaves.

## Conclusions

Mechanical stress has been used in farming for a long-time to harden crops and promote stress acclimation [1, 16]. The mechanism by which MS can promote stress tolerance and defense responses is not well understood. Genome-wide transcriptome analysis shows that a short period of MS can enhance the expression of several genes implicated in stress responses [9, 10, 12, 71]. Here, we showed that a short period of repetitive MS to juvenile seedlings promoted resistance and primed defense gene expression upon subsequent challenge with pathogens via JA signalling. Prior exposure of plants to MS altered cell wall appositions and defense-related metabolites that can be attributed to the enhanced resistance against *A. brassicicola*. The altered gene expression in cauline leaves in the absence of further MS supported previous reports implicating epigenetic processes in facilitating thigmomorphogenesis [21, 24, 65]. Understanding the factors (the duration, intensity, the age of the plant as well as signalling pathways and genome-wide regulation) that enable MS acclimation responses in plants could help growers to utilise MS to induce defense mechanisms in greenhouse propagated seedlings before transplanting them into the field.

## Methods

### Plant materials and growth conditions

The JA-insensitive mutant *coi1–16* (CS68788) was obtained from TAIR and *coi1–21* (SALK_035548) was obtained from Prof. Jose Ramon Botella (University of Queensland, Australia) [72]. Both *coi1* alleles have a Col-0 wild-type background and were supplied as homozygous seed stock lines with the same gene locus (AT2G39940). *coi1* mutant alleles have a characteristic male sterile phenotype and will not set seeds when grown at normal growth temperatures [73]. However, when *coi1* mutant alleles are grown at 16 °C male fertility is restored and seeds set normally as previously described [74]. We assessed the phenotypes of *coi1–16* in comparison to *coi1–21*, confirming that all siblings from both germplasms displayed a male sterile phenotype and displayed no notable differences in growth or morphology. However, when these alleles were grown in the cold, we got more seeds from *coi1–16* and hence selected this germplasm for subsequent studies described in this paper. There were no other obvious differences in phenotypes of the two *coi1* alleles when grown in the cold (data not shown).

Seeds were grown in sterilized seed raising mixture soil (DEBCO Pty, Australia) with slow-release fertilizer (Osmocote, Garden City Plastics, Australia) and stratified at 4 °C for 2–3 days before being transferred to the growth chamber at 22 °C with 16 h-light/8 h-dark photoperiods with a relative humidity of 60% and a light intensity between 140 and 150 μmol m^− 2^ s^− 1^. Plants were watered from the bottom of growth trays once every week.

### Mechanical stimulation of plants

Juvenile plants (10–12 days old after growth, DAG) were gently stimulated by touching the rosettes clockwise and anticlockwise for 8–10 s with a very soft brush to constitute a single MS. Care was taken not to break the leaves as this could result in a wound-induced response. For repetitive mechanical stress, touch was done twice daily (with 8 h intervals) for 7-days (MS) unless otherwise stated. After 7-days of mechanical stimulation, images of both touch and untouched plants were taken for analysis of thigmomorphogenetic traits (rosette area, petiole length, and inflorescence height) with ImageJ software (https://imagej.nih.gov/ij/download.html). The plant height measured corresponds to the main floral bolt.

### Pathogen assays

Pathogen culture and plant infection were performed as previously described [72, 75]. Pathogens used for the experiment were *A. brassicicola* (isolate DAR 27028) and *Botrytis cinerea* (DAR 77536) both were obtained from the Department of Primary Industries, NSW (http://www.dpi.nsw.gov.au). The pathogens were grown on potato dextrose agar (PDA) plate for about 12–14 days at 23 °C. The *B. cineria* and *A. brassicicola* matured spores showed visible black and brown colouration respectively on PDA plate. Spores were washed from the plate with distilled water, filtered with miracloth and counted with a haemocytomer. Test plants were covered with a transparent plastic dome to increase humidity the day before inoculation as previously described [34]. Plants were drop-inoculated with 5 μl of 3.5 × 10^4^ spores/mL of *B. cinerea* or *A. brassicicola* and disease symptoms scored for necrotic spots and chlorosis. Disease progression was analysed by taking images of leaves and the necrotic lesion area was measured with Image J software (https://imagej.nih.gov/ij/download.html).

### RNA extraction, cDNA synthesis, and gene expression analyses

Leaf tissues were collected at indicated time point and quickly frozen in liquid nitrogen. Samples were grounded with TissueLyser (Qiagen) in a 2 mL Eppendorf tube containing two steel balls. RNA for gene expression analysis was extracted using Spectrum Plant Total RNA Kit (Sigma) following the manufacturers’ protocol. First-strand cDNA was made using Transcriptor First Strand cDNA Synthesis Kit (Roche). Quantitative RT-PCR was performed using a Roche Light Cycler 480 (http://www.roche.com) with FastStart DNA Master SYBR Green I kit (Roche) as described in the manual. Primers were designed across exon junction using the online Primer3Plus program (http://www.bioinformatics.nl/cgi-bin/primer3plus/primer3plus.cgi). Secondary structures were identified to eliminate primer-dimers using the IDT UNAFOLD program (https://sg.idtdna.com/UNAFold). Gene expression levels relative to the previously validated reference gene *β-ACTIN2* (At3g18780) were used for each cDNA sample following the Pfaffle method [76]. Only *ACT2* was used because it did not change its transcription in response to MS in our experimental setup. Specific primer sequences for analysis are listed in Table 1. Primer quality and efficiency were checked by creating serial dilution and a standard curve for each primer to correctly determine amplification efficiency and to remove bad primers. Primers had an efficiency range between 90 and 100%.

**Table 1:** List of qPCR primers

Primer name	Function		Primer sequence (5′-3′)	Locus
PR1	Induced by SA	F	TGCAGTGGGACGAGAGGGT	AT2G14610
		R	CCACATGTTCACGGCGGAGA	
RBOHD	Response to ROS	F	GGTTTCAACGCCTTTTGGTA	AT5G47910
		R	CGGTTTGATGCTTGATCTGA	
PDF1.2	ET/JA response	F	GTTGCATGATCCATGTTTGG	At5g44420
		R	CACCCTTATCTTCGCTGCTC	
ACTIN 2	Reference gene	F	TGTCGCCATCCAAGCTGTTCTCTCC	AT3G18780
		R	ACGTCCAGCAAGGTCAAGACGGAGG	
GSL6	Callose deposition	F	CAAACCATGACGCTCCATAA	AT1G05570
		R	GGTTCGTGGTATCACCCTTG	
PRX71	Stem lignification	F	TTGTTGAACGGCAACGGAGTCACG	AT5G64120
		R	AGCCGCTCGTGACACAGTCATTCT	
OPR3	JA pathway	F	ACGTGGGAACCATCGGGCAACAAA	At2g06050
		R	AGCAAGTTGTGGAAGCAGTTCACGC	
VSP1	JA signalling	F	GGATCGAAGTTGACGCAAGTG	AT5G24780
		R	CTCAACCAAATCAGCCCATTG	
PAD3	Camalexin biosynthesis	F	TTCCTCTGTTTCCTCGTCCT	AT3G26830
		R	ATGATGGGAAGCTTCTTTGG	
GST1	Glutathione binding	F	TAATAAAAGTGGCGATGACC	AT1G02930
		R	ACATTCAAATCAAACACTCG	
TCH3	Calcium signalling	F	AGCCTTCCGCGTATTCGACAAGA	AT2G41100
		R	CCGTCACCATCTGCATCCGC	
TCH4(XTH22)	Cell wall modification	F	TGTCTCCTTTGCCTTGTGTG	AT5G57560
		R	GAAACTCCGCAGGAACAGTC	
ERF2	Response to ET	F	TGAGGTTAATTCCGGTGAACC	AT3G23240
		R	TCAACTTCCCGTTTTCAGACGA	

### Histochemical analysis

Reactive oxygen species (ROS) accumulation was determined as previously described [77]. Briefly, infected and control leaves were vacuum infiltrated with 0.1% (w/v) Diaminobenzidine (DAB) (Sigma) (pH 3.9) for 3 min and repeated if not all leaves were infiltrated. Leaves were placed on a moist filter paper in a Petri dish under high humidity until a brown precipitate was observed (3–5 h) and cleared in 96% (v/v) ethanol and then fixed with a solution of 3:1:1 ethanol/lactic acid/glycerol and observed under a light microscope (Zeiss Stemi 2000C mounted with Axiocam).

For necrotic lesions, leaves were stained with lactophenol-trypan blue solution [78] (20 mL phenol, 20 mL lactic acid, 40 mL glycerol, 20 mL water and 0.05% trypan blue mix and 200 mL 96% ethanol) for 2 min and de-stained in chloral hydrate (2.5 g in 1 mL of 30% glycerol) overnight. Leaves were viewed under a Carl Zeiss Axio Scope A1 microscope equipped with interference or phase-contrast optics and digital camera. The lesion area for each sample was determined using the ImageJ software. The scale for images was input into the software and using the freehand tool, region of necrotic lesion was selected and measured (W.S. Rasband, National Institutes of Health; http://imagej.nih.gov/ij/).

Lignin deposition was determined as follows; MS stressed, and control leaves were destained in 100% ethanol. Leaves were transferred into freshly prepared 2.5% (w/v) phloroglucinol in 75% ethanol at room temperature and then mounted in 33% HCl for 5 min.

Callose deposition was determined by destaining leaves in 95–100% ethanol to remove chlorophyll. After 24 h, leaves were washed in 0.07 M phosphate (pH 9.0) buffer and then transferred into a new 0.07 M phosphate buffer (pH 9.0) containing 0.05% aniline blue (Sigma) for 2 h in the dark. Samples were visualized and photographed with Carl Zeiss Axio Scope A1 microscope equipped with fluorescent UV light (excitation 340 to 380 nm, emission 425 nm). The calculation of the aniline blue-emitted fluorescence of callose deposits was determine by using the ImageJ software (W.S. Rasband, National Institutes of Health; http://imagej.nih.gov/ij/). “The freehand selection tool” was used to define the area of callose deposit. Note, all images must have the same camera settings (scale/focal length and lightening) during capturing. For set measurement, the area integrated intensity and mean grey value were selected in program and the analyse tool ROI manager was used to select and measure several callose depositions per image. Background was selected for every image determined by area with no fluorescence to determine the corrected total fluorescence intensity for callose deposit.

### Phytohormone quantification

For quantification of the endogenous JA hormone, the whole rosette was excised and quickly placed in liquid nitrogen and transferred to − 80 °C freezer for further analysis. Extraction of hormones was performed as previously described with modifications [79, 80] using the UPLC/ESI-MS/MS (Water, Milford, USA) at the Western Sydney University, Mass Spectrometry Facility, Campbelltown, Australia. The pure JA standard hormone was obtained from Sigma Aldrich. The internal standard- deuterated compound [^2^H_4_]-SA (C_7_H_2_D_4_O_3_) was obtained from Olchemim Ltd., Olomouc, Czech Republic and the jasmonic-d_5_ acid (C_12_H_13_D_5_O_3_) was obtained from CND isotopes, Canada. The whole plant was excised for analysis to reduce variation resulting from leaf age. In total, 10–15 biological replicates each containing 2 plants were used for extraction.

Briefly, 200 mg of fine power from tissue ground in liquid nitrogen was put in a 2 mL Eppendorf tube. 1 mL of extraction solvent (methanol: water: acetic acid, 69:30:1 (v/v/v) containing 100 ppb final concentration of the internal standard (a labelled form of the compound ^2^H_4_ SA, ^2^H_2_ JA) was added. The control (blank) contained extraction solvent with the internal standard without plant materials. Samples were vortex at 4 °C for 5 min. After centrifugation (10,000 rpm for 15 min at 4 °C), the supernatant was collected, and the pellet was re-extracted with 0.5 mL of extraction solvent and the extraction repeated twice. The upper organic layers collected, combined and evaporated to dryness under nitrogen gas. Each sample was resuspended in 250 μl of methanol: water (70:30, v/v) by ultrasonication for 15 min (4–8 °C) and filtered through a 0.22 μm PTFE filter (Waters, Milford, MA, USA). Samples (10 μl) were then analysed by UPLC/ESI-MS/ MS. For the analysis of the extracts, a HALO™ C18 (Advanced Materials Technology, Inc., Wilmington, USA) column (2.1 × 75 mm, 2.7 μm) was used. A calibration curve was created for quantification containing each of the unlabelled analyte pure compounds (JA) from 1 ppb to 200 ppb. To each standard solution, the same amount of internal standard was added. Calibration curve for each analyte was generated using MassLynx 4.1™ software (Waters, USA).

### Statistical analysis

Significant differences between control and MS plants were analysed using analysis of variance (ANOVA) according to Bonferroni post hoc test (*P* < 0.05) for more than 2 data sets, using SigmaPlot 14.0 software. On some data, where only 2 groups were compared, the Student’s *t*-test was used. Error bars show standard error (SE).

## Data Availability

The datasets used and/or analysed during the current study are available from the corresponding author on reasonable request. The datasets utilising genetic polymorphisms and DNA sequence information used in this study are available from TAIR [https://www.arabidopsis.org].

## Abbreviations

MS: Mechanical stress
JA: Jasmonic acid
SA: Salicylic acid
TCH: Touch
WT: Wild type
